# Outcomes of dual-mobility acetabular cup for instability in primary and revision total hip arthroplasty

**DOI:** 10.1007/s10195-014-0324-9

**Published:** 2014-10-21

**Authors:** Riazuddin Mohammed, Keith Hayward, Sanjay Mulay, Frank Bindi, Murray Wallace

**Affiliations:** 1 Wrightington Hospital, Appley Bridge, Wigan, WN6 9EP UK; 2 Queens Hospital, Burton Hospitals NHS Foundation Trust, Burton on Trent, DE13 0RB UK

**Keywords:** Hip arthroplasty, Dislocation, Instability, Dual-mobility socket

## Abstract

**Background:**

The concept of a dual-mobility hip socket involves the standard femoral head component encased in a larger polyethylene liner, which in turn articulates inside a metal shell implanted in the native acetabulum. The aim of this study was to assess outcomes from using a Serf Novae^®^ Dual Mobility Acetabular cup (Orthodynamics Ltd, Gloucestershire, UK) to address the problem of instability in primary and revision total hip arthroplasty (THA).

**Materials and methods:**

A retrospective review was carried out of all hip arthroplasties performed in a District General Hospital utilising the dual-mobility socket from January 2007 to December 2012. Clinical and radiological outcomes were analysed for 44 hips in 41 patients, comprising 20 primary and 24 revision THA. The average age of the study group was 70.8 years (range 56–84 years) for primary and 76.4 years (range 56–89 years) for revision arthroplasty. Among the primary THA, always performed for hip osteoarthritis or in presence of osteoarthritic changes, the reasons to choose a dual mobility cup were central nervous system problems such as Parkinson’s disease, stroke, dementia (10), hip fracture (5), failed hip fracture fixation (2), severe fixed hip deformity (2) and diffuse peripheral neuropathy (1). The indications for revisions were recurrent dislocation (17), aseptic loosening with abductor deficiency (4), failed hemiarthroplasty with abductor deficiency (2) and neglected dislocation (1).

**Results:**

At a mean follow-up of 22 months (range 6–63 months), none of the hips had any dislocation, instability or infection and no further surgical intervention was required. Radiological assessment showed that one uncemented socket in a revision arthroplasty performed for recurrent dislocation had changed position, but was stable in the new position. The patient did not have complications from this and did not need any surgical intervention.

**Conclusions:**

Even though postoperative hip stability depends on several factors other than design-related ones, our study shows promising early results for reducing the risk of instability in this challenging group of patients undergoing primary and revision hip arthroplasty.

**Level of evidence:**

IV.

## Introduction

Total hip arthroplasty (THA) is a very successful surgical intervention for advanced arthritis, but is a surgical challenge in patients with compromised abductor mechanism or systemic conditions that make them more prone to instability. Revision THA for recurrent dislocation is a significant challenge for both the patient and the surgeon to manage. Numerous surgical and patient-related factors have been implicated in the aetiology of prosthetic dislocation [1]. Various surgical options in dealing with instability include constrained liners, liner augments, trochanteric advancement, large-diameter prosthetic heads and dual articular sockets.

**Fig. 1:**
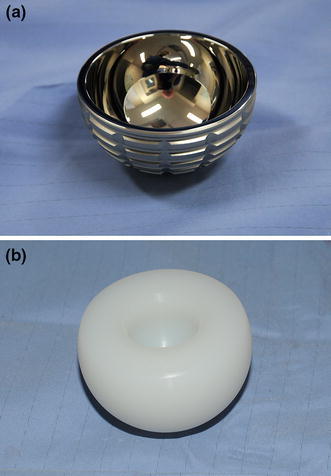
**a**, **b** Serf Novae^®^ Dual Mobility uncemented acetabular socket components

The concept of a dual-mobility hip socket involves the standard femoral head component captured in a larger polyethylene liner, which in turn articulates inside a metal shell implanted in the native acetabulum. This large-diameter articulation increases the primary arc range and the lever range, thereby improving the range of movement and the stability. The aim of this study was to analyse the complications and outcomes of the Serf Novae^®^ Dual Mobility Acetabular cup (Orthodynamics Ltd, Gloucestershire, UK) used to address the problem of instability in primary and revision THA (Fig. 1a, b).

## Materials and methods

**Fig. 2:**
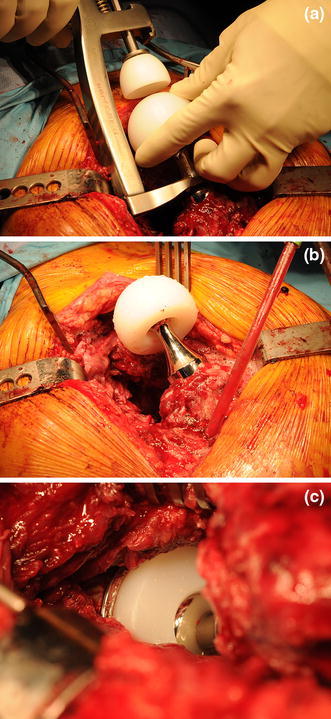
**a**, **b**, **c** Intra-operative clinical photographs of socket-only revision being performed

A retrospective review was carried out of all hip replacements performed from January 2007 to December 2012 in a District General Hospital in the United Kingdom utilising a dual-mobility socket. Patients were identified from the theatre database and their clinical data together with follow-up radiographs were analysed. All the procedures were performed under antibiotic prophylaxis and in a laminar flow operating theatre by experienced hip arthroplasty consultants. Depending on the surgeon’s preference, a modified lateral approach or a posterior approach to the hip joint was used (Fig. 2a–c). All patients received prophylactic intravenous antibiotics for 24 h postoperatively. Venous thromboprophylaxis was with low-molecular-weight heparin in the hospital and discharge with rivaroxaban for 5 weeks. This regimen is based on a standardised departmental policy and is based on the guidelines issued by the National Institute for Health and Care Excellence, UK.

Standard postoperative rehabilitation as for any THA was followed. Patients were followed up at 6 weeks, 6 months, 1 year and then 3 yearly after the THA. Data was collected regarding patient demographics, indications for arthroplasty, complications and implant survival.

Primary outcome measures analysed the incidence of dislocation and the necessity for any surgical intervention for dislocation. Secondary end-points included infection, peri-prosthetic fractures, radiological assessment of implant position and evidence of loosening.

Five patients were lost to follow-up. Two patients who underwent arthroplasty for proximal femur fracture died within 6 weeks after the procedure. Three patients did not attend the first post-discharge clinic. In total, 44 hips (20 primary and 24 revision THAs) in 41 patients were available for analysis.

In the primary THAs, the femoral component was un-cemented hydroxyapatite-coated modular titanium alloy stem in 8 hips and a polished double-tapered cemented femoral component in 12 hips. The mean time between primary and revision arthroplasty was 12.8 years (range 10 months–23 years). Nineteen of the 24 revision THAs involved socket-only revision. Of the five hips undergoing full revision, two hips had an un-cemented hydroxyapatite-coated modular titanium alloy stem and three had a polished double-tapered cemented femoral component. Seven of the dual-mobility cups were cemented (all in the revision group) and the remainder were hydroxyapatite-coated un-cemented cups. The details of the patient groups, indications and component sizes are depicted in Table 1.

**Table 1:** Details of patients included in the study

	Primary THA	Revision THA
Number of hips	20	24
Number of patients	17	24
Mean age in years (range)	70.8 years (56–84)	76.4 years (56–89)
Gender (M/F)	8/9	7/17
Right side/left side	9/11	12/12
Indications for dual mobility socket use:		
	Central nervous system problems such as Parkinson’s disease, stroke, dementia: 10	Recurrent dislocation: 17
	Hip fracture: 5	Aseptic loosening with abductor deficiency: 4
	Failed hip fracture fixation: 2	Failed hemiarthroplasty with abductor deficiency: 2
	Severe fixed hip deformity: 2	Neglected dislocation: 1
	Diffuse peripheral neuropathy: 1	

Primary THA was performed in five patients leading active lifestyles with fracture of the neck of femur, who had coexistent arthritis in the hip. In two patients with failed internal fixation for proximal femur fracture and whose hip joints had secondary osteorthritis, primary THA with dual-mobility socket was performed to address potential instability, as would be anticipated in a hip fracture scenario.

## Results

**Fig. 3:**
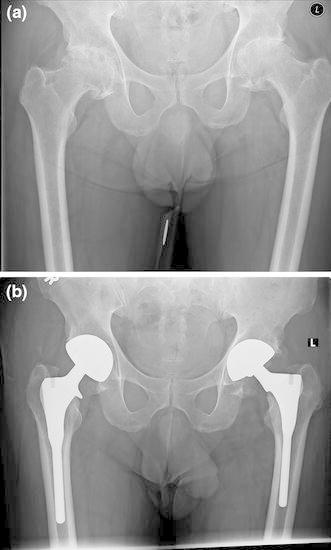
**a**, **b** Pre- and post operative radiographs of staged bilateral dual-mobility socket primary THA in a 73-year-old male with severe Parkinson’s disease

At a mean follow-up of 22 months (range 6–63 months) there were no dislocations in any of the hips in either group (Fig. 3a, b). None of the patients had any other complications such as infection, neuro-vascular injury or peri-prosthetic fracture. No further surgical intervention was required for any patient.

**Fig. 4:**
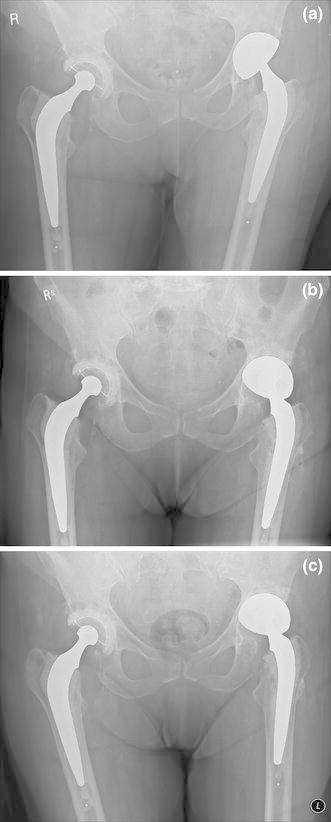
**a** Immediate postoperative radiograph of an 86-year-old female’s revision THA for recurrent dislocation showing satisfactory socket position. **b** 6 months postoperative radiographs depicting change in the socket orientation. **c** Radiograph at 30 months postoperative period with no further change in cup position

Radiological assessment showed that 42 hips (95 %) had an abduction angle in the acceptable range of 35°–50°. Anteversion was much more difficult to measure as the radiographs were not standardised. One uncemented socket in the revision group performed for recurrent dislocation had changed position at the 6-month review, but was stable in the new position (Fig. 4a–c). The patient did not have any complications from this and no intervention was necessary. No other radiographic complications were noted in any of the remaining acetabular components.

## Discussion

The dual-mobility concept in THA was developed in the 1970s by Gilles Bousquet and André Rambert from France. The idea was to combine the “low-friction arthroplasty” principle of Charnley together with the advantage of a big femoral head principle of MacKee. The initial design of the cup had tripod fixation points on the rim of the shell along with an alumina ceramic outer coating. The newer-generation sockets have a dual-layer hydroxyapatite and alumina ceramic coating to enhance bony in-growth [2]. The shell is hemispherical with 0.5-mm polar effacement for better seating in the native acetabulum. The polyethylene insert is modified with a chamfer margin to reduce impingement on the neck of the femur prosthesis.

The Serf Novae^®^ Dual Mobility Acetabular cup (Orthodynamics Ltd, Gloucestershire, UK) has the advantages of increased range of movement (up to 186°), better coaptation between the components and less stress on the bone implant interface, and is available in a range of sizes (43–69 mm for un-cemented, 43–63 mm for cemented fixation) and has options for cemented, cementless or reconstructive surgery. It can be used with either a 22.25-mm or a 28-mm femoral head.

One of the potential disadvantages levelled against dual-mobility articulation is the theoretical increased risk of polyethylene wear, because both the concave and convex surfaces of the polyethylene liner articulate with the metal components. However, a retrieval analysis study of 40 dual-mobility sockets showed that both the mean total wear (the sum of the wear on the convex and concave surfaces) as well as the mean annual total wear volume of the polyethylene liner was not more than that for conventional metal–polyethylene bearings [3].

A unique complication of the dual-mobility socket is intra-prosthetic dislocation (IPD) [4]. In this scenario, the femoral head dislodges from the mobile polyethylene liner. The metal head can then articulate with the metal socket, leading to devastating complications, including severe metallosis [5]. We did not encounter IPD in our series, probably because this complication is usually seen in the medium term, at about 8–10 years or more after the THA.

The indication for using a dual-mobility socket was quite varied in our series of patients and it was chosen for primary THA if the patient was deemed to have a higher risk of dislocation, supported by pre-operative clinical examination findings, or if an intra-operative query arose about potential instability. In the revision scenario, dual-mobility cups were used for established instability with or without aseptic loosening of the socket. The success of the socket in preventing dislocation in our series is in keeping with its versatility in various indications like hip trauma, primary hip arthroplasty and revision THA [6–8].

In our series, none of the patients had any subsequent instability or further surgical intervention. Similar short-term follow-up published evidence also supports very low dislocation rates and excellent implant survival rates [9–11]. Ten patients in our cohort had the procedure through a posterior approach to the hip joint. Studies have shown that meticulous surgical technique with careful soft tissue repair is essential to avoid instability, irrespective of the approach used [12].

Our study supports the concept of dual-mobility acetabular components in preventing the risk of dislocation for both revision THA, where instability is the main or associated reason for revision, and also in primary THA, where the dislocation can be a potential problem. The hip arthroplasty load in our centre is approximately 300–350 primary THAs and 10 revision THAs per year. The dual-mobility socket is currently the implant of choice and the only implant used in our unit in this challenging group of patients.

It is interesting that our cohort of 24 revision THAs included 19 hips with acetabular component-only revision. Revision involving the socket only can be utilised if the femoral component is well fixed and in satisfactory alignment, and thereby not a contributor to the instability. Civinini et al. [13] have shown that at 3-year mean follow-up, dual-mobility cups reduce dislocation for isolated acetabular revisions, without increased risk of loosening. Though limited by small numbers and short follow-up, our data show that one component-only revision can be successful in the management of prosthetic hip instability, thereby avoiding the complications of major revision hip surgery.

Our study is limited by being a retrospective series with a small number of patients and short follow-up. However, the study is an independent series from a heterogeneous district hospital setting outside mainland Europe.

Even though prosthetic hip stability depends on many factors other than the implant-related ones, the good results shown in our study reinforce the excellent outcomes reported in the literature, in both primary and revision THA, for the efficacy of dual-mobility cups in managing hip dislocation.
